# Supplementary material for: Key Process and Factors Controlling the Direct Translocation of Cell-Penetrating Peptide through Bio-Membrane
Source: Int J Mol Sci. 2020 Jul 30;21(15):5466. doi: 10.3390/ijms21155466 (PMC7432884; doi:10.3390/ijms21155466)

Suppl. Figure 1

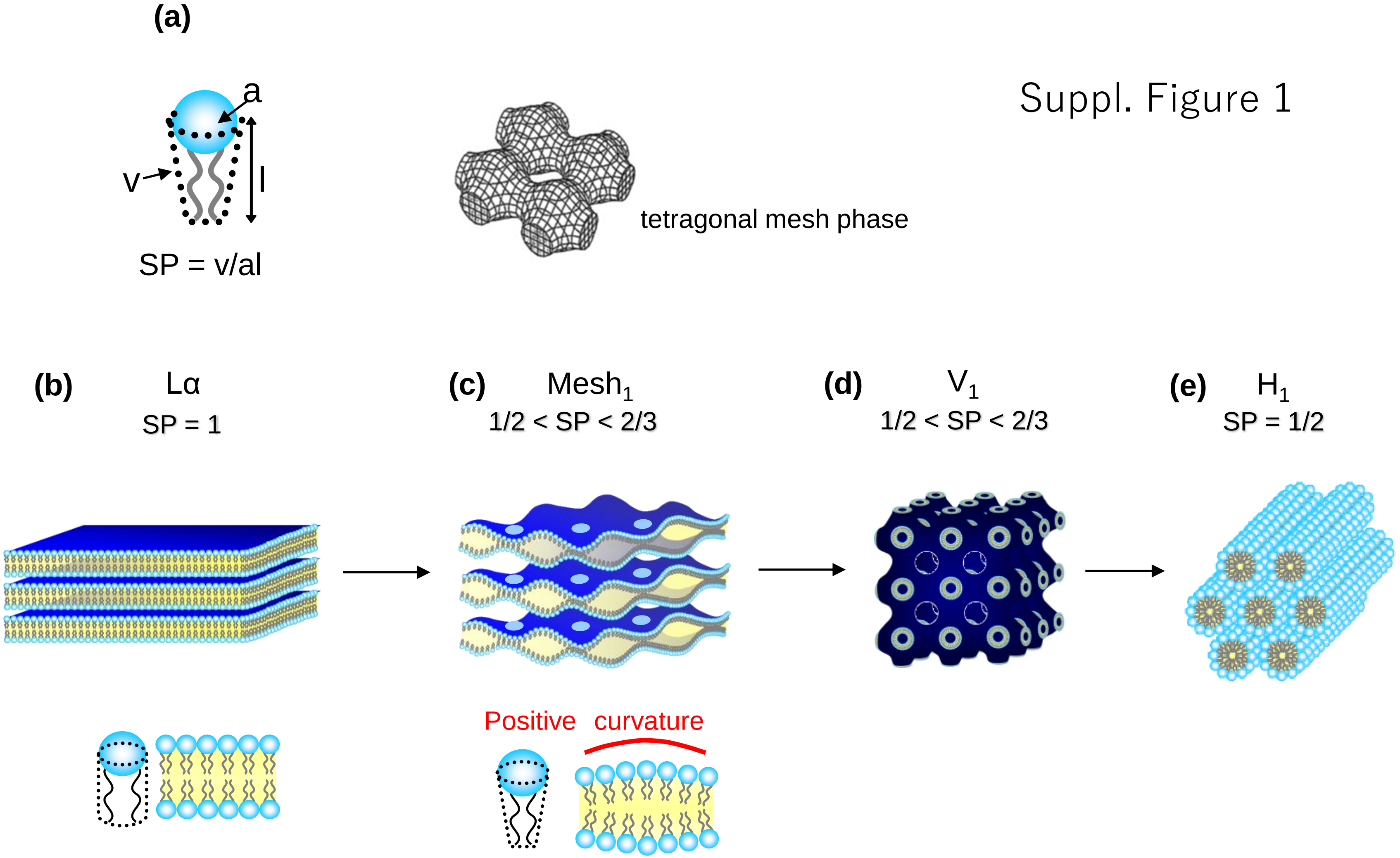

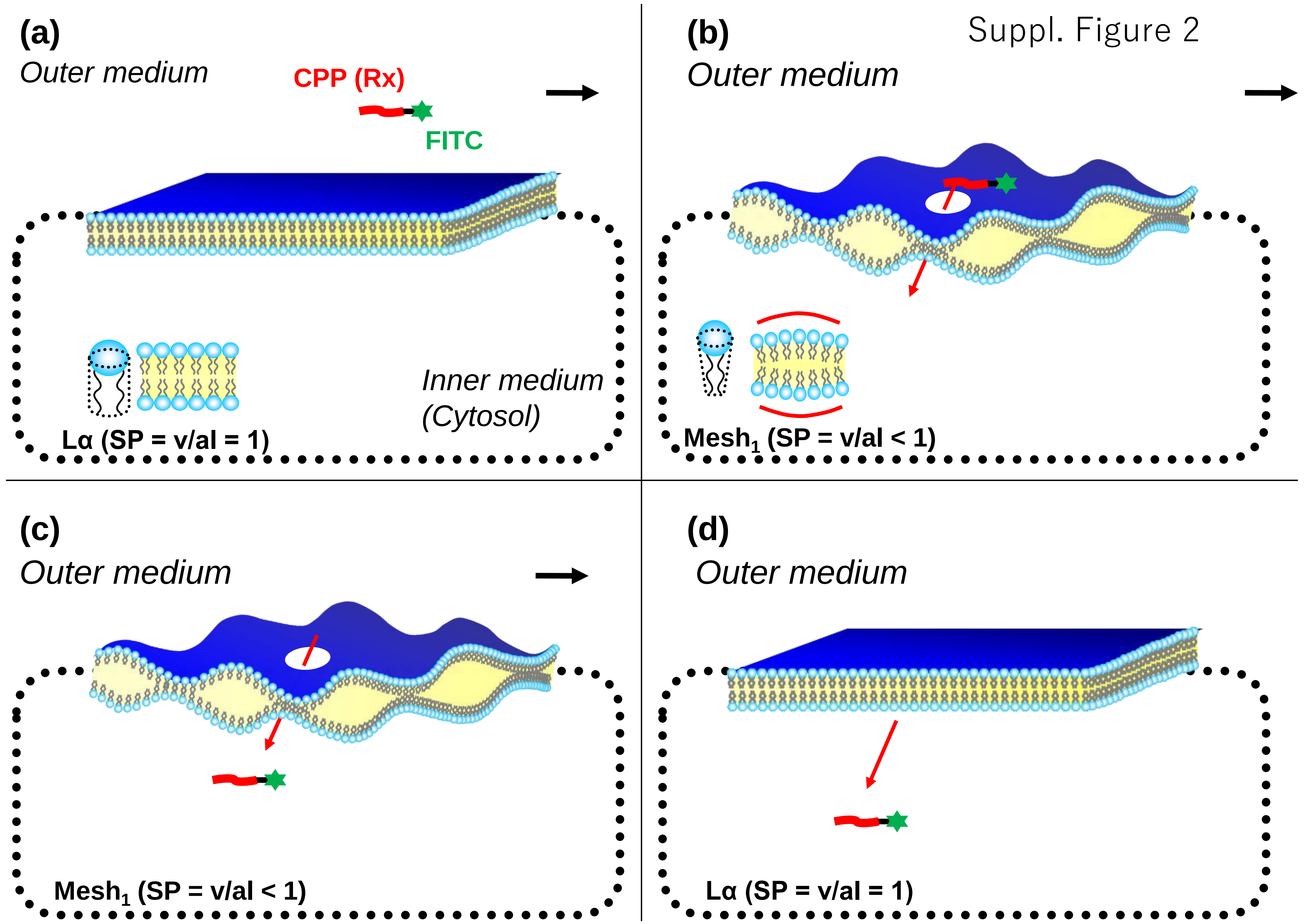

Suppl. Figure 2

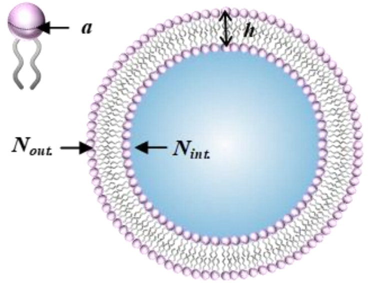

Suppl. Figure 3

The number of phospholipid molecules of the outer surface of the vesicles (N_{out}) is represented by the formula (3).

$$N_{out} = \frac{4\pi\left(\frac{d}{2}\right)^2}{a} \quad (3)$$

The number of phospholipid molecules of the internal surface of the vesicles (N_{int}) is represented by the formula (4).

$$N_{int} = \frac{4\pi\left(\frac{d}{2} - h\right)^2}{a} \quad (4)$$

Since the molecular occupation area a of E-PC is 0.71nm^2 and the thickness the bi-layer formed by the phospholipid is about 5nm, the number of phospholipid molecules consisting GUV of E-PC is represented by the following formula (5).

$$N_{tot} = \frac{\left[4\pi\left(\frac{d}{2}\right)^2 + 4\pi\left(\frac{d}{2} - 5\right)^2\right]}{a} \quad (5)$$

Since the diameter of GUV is about 14μm, N_{tot}=1.7 X 10^9 by formulae (5), N_{out}=8.8 X 10^8 from formulae (4) and N_{int}=8.8 X 10^8 are obtained.

The number of the vesicles N_{vesic} in 1mL vesicle suspension is represented by the following formula (6).

$$N_{vesic} = \frac{M_{lipid} \times N_A}{N_{tot} \times 1000} \quad (6)$$

Wherein M_{lipid} and N_A represent molar concentration (mol/L) of the phospholipid and the Avogadro number (6.0 X 10^{23}(mol^{-1})), respectively.

Since the molar concentration of the phospholipid is 1.7mM, N_{vesi} is obtained by the above formula (6) as follows.

N_{vesi} =5.82 X 10^8 (unit/mL).

Suppl. Scheme1

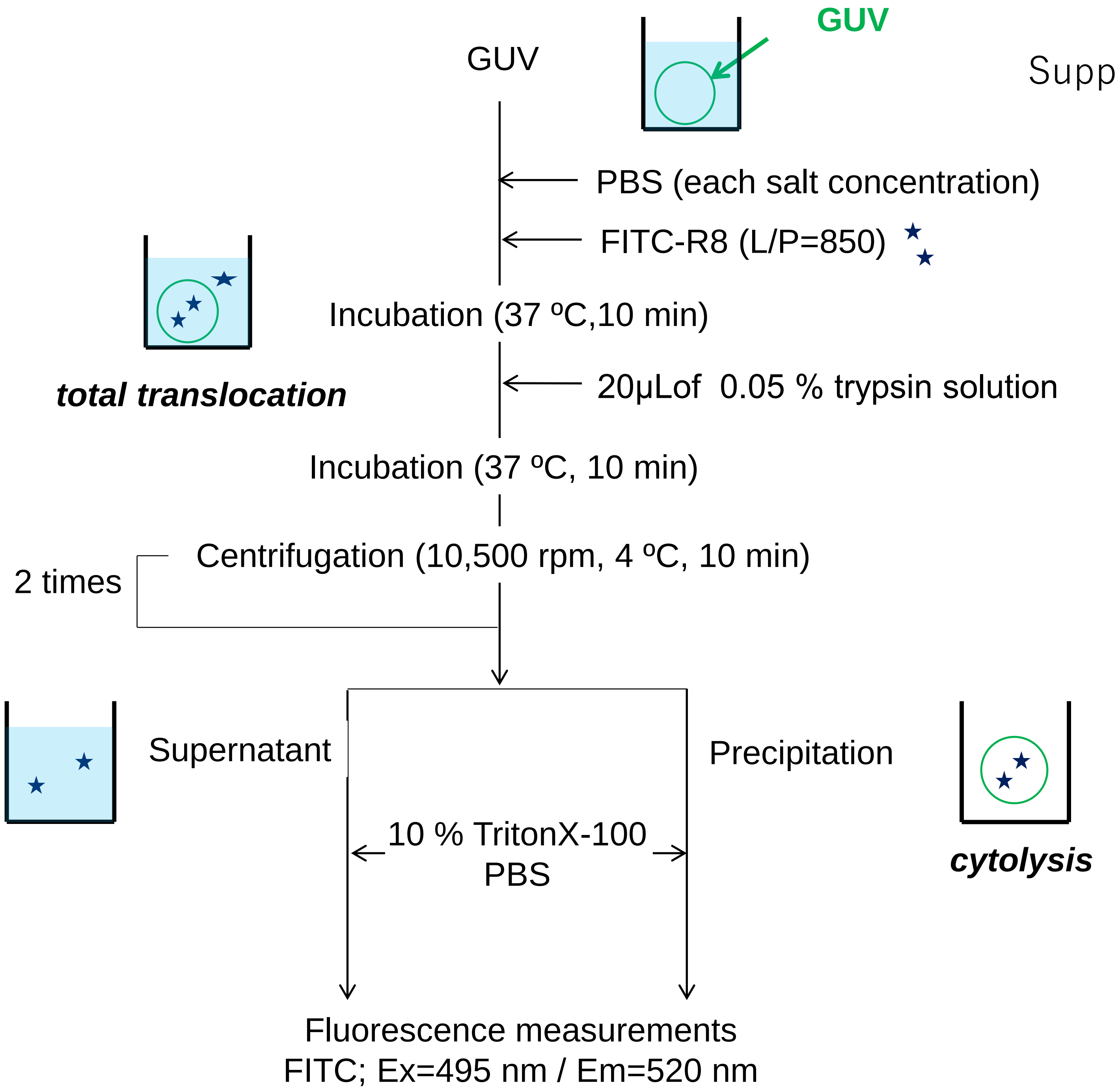

Supplement: Supplementary file 1 [file ijms-21-05466-s001.pdf]
